# Characterization and Comparison of *Clavibacter michiganensis* subsp. *nebraskensis* Strains Recovered from Epiphytic and Symptomatic Infections of Maize in Iowa

**DOI:** 10.1371/journal.pone.0143553

**Published:** 2015-11-24

**Authors:** Azeem Ahmad, Gladys Y. Mbofung, Jyotsna Acharya, Clarice L. Schmidt, Alison E. Robertson

**Affiliations:** Department of Plant Pathology and Microbiology, Iowa State University, Ames, Iowa, United States of America; University of Nebraska-Lincoln, UNITED STATES

## Abstract

*Clavibacter michiganensis* subsp. *nebraskensis* (*Cmn*), the causal organism of Goss’s wilt and leaf blight of maize, can be detected in the phyllosphere of its host prior to disease development. We compared the morphology and pathogenicity of 37 putative isolates of *Cmn* recovered from asymptomatic and symptomatic maize leaves. Thirty-three of the isolates produced mucoid orange colonies, irrespective of the source of isolation and all but four of these isolates were pathogenic on maize. The remaining 4 isolates recovered from asymptomatic leaves had large fluidal yellow colonies, and were non-pathogenic on maize. Isolates varied in their aggressiveness on a susceptible hybrid of maize but no significant differences in aggressiveness were detected between epiphytic isolates and those recovered from diseased maize tissues. The genomics of *Cmn* is poorly understood; therefore as a first step to determining what genes may play a role in virulence, we compared 33 putative virulence gene sequences from 6 pathogenic and a non-pathogenic isolate recovered from the phyllosphere. Sequence polymorphisms were detected in 5 genes, cellulase A, two endoglucanases, xylanase B and a pectate lyase but there was no relationship with pathogenicity. Further research is needed to determine what genes play a role in virulence of *Cmn*. Our data show however, that the virulence factors in *Cmn* likely differ from those reported for the closely related subspecies *michiganensis* and *sepedonicus*.

## Introduction

Goss's wilt and leaf blight of maize, caused by *Clavibacter michiganensis* subspecies *nebraskensis* (*Cmn*), is economically important in the United States (US) [1]. The disease was first observed affecting maize plants in south central Nebraska in 1969 and soon after was reported from the seven surrounding states [2, 3]. After decades of reduced incidence, the disease recently reemerged throughout the Midwest [1, 4–6] Texas, [7, 8], Louisiana [9, 10], and in Canada [3, 8, 11]. Significant yield losses of up to 50% have been recorded, and usually depend on the crop developmental stage at which the maize hybrid was infected [12].

*Cmn* can infect maize at any crop developmental stage causing either leaf blight symptoms and/or wilting of the plant. Goss’s leaf blight is more common and is characterized by large elongated leaf lesions with characteristic small (<1mm^2^) dark green to black water-soaked spots in the periphery of the lesions. Wilt occurs when the bacteria in the vasculature becomes systemic and is characterized by an orange to brownish discoloration of the internal vascular tissue of the stalk and stunting of the maize plant [5]. The wilting is more common on young seedlings and when seed-transmission of the pathogen occurs

Although infected seed can be a source of inoculum [13,14,15], the main source of inoculum for Goss’s wilt and leaf blight is surface *Cmn*-infested residue within which the bacterium is able to survive for at least 10 months [5, 16]. Smidt and Vidaver [2] detected *Cmn* on asymptomatic corn leaves in the field and suggested an epiphytic phase for the pathogen that may contribute to disease development. However, limited research has been done to examine this hypothesis. In several other pathosystems, epiphytic bacterial are important in the epidemiology of the disease but the importance of epiphytic *Cmn* in Goss’s wilt and leaf blight development is unknown.

The genomics of *Cmn* is poorly understood. Three of the 5 *michiganenisis* subspecies genomes have been sequenced and recent comparative genome analyses have shown that the genome of *Cmn* was very similar to that of *Cmm* and *Cms* [17, 18]. In the subspecies *michiganensis* and *sepedonicus*, major virulence factors responsible for disease induction were plasmid borne while the genes responsible for successful host colonization were chromosomally encoded. The chromosomal virulence factors were identified as serine proteases of the Chp and Ppa family, and a tomatinase A gene, all located on an area of chromosome known as a pathogenicity island [13]. The plasmid-encoded factors were identified as a pat-1 serine protease and a cellulase A gene [18]. Vidaver [2] reported plasmids were not required for pathogenicity in *Cmn*. Consequently, Eichenlaub and Gartemann [17] suggested that virulence mechanisms in *Cmn* may be different from those observed in *Cmm*. Currently, little is known regarding virulence factors of *Cmn*.

The goals of this study were (i) to compare the morphology and pathogenicity of isolates of *Cmn* recovered from the phyllosphere of apparently healthy maize leaves with isolates recovered from maize leaves with characteristic Goss’s leaf blight symptoms, and (ii) to assess sequence heterogeneity between a non-pathogenic isolate of *Cmn* and pathogenic isolates in 33 genes that encode for putative virulence factors.

## Materials and Methods

### Bacterial strains and morphological characterization

A total of 37 putative *Cmn* strains were evaluated in this study (Table 1). Ten of the isolates were recovered from corn plants with symptoms of Goss’s leaf blight in 3 fields in Iowa. Briefly, a piece of leaf tissue was cut using a sterile razor blade from the leading edge of a lesion, surface disinfested in 0.525% NaOCl, rinsed in sterile distilled water and placed on CNS medium [15, 19], which is semi-selective for *Cmn*. The remaining twenty-seven strains were recovered from washes of leaves harvested in 2012 and 2013 from apparently healthy corn plants grown in minimally tilled fields with a history of Goss’s disease in the previous growing season in Iowa (6 fields) and Nebraska (1 field). All culture plates were incubated at 25°C for a minimum of 5 days. Colonies with morphological characteristic of *Cmn* are typically apricot-orange, small (3–5 mm in diameter) circular, convex, glistening and butyrous with entire margins after 5 to 7 days of growth on Nutrient Broth Yeast Extract (NBY) medium at 22 to 26°C [2]. Putative colonies of *Cmn* were sub-cultured on NBY medium and incubated under the same conditions. The resultant colonies were streaked on NBY plates a second time to obtain single colonies. A single colony from each plate was used to make inoculum for pathogenicity studies, genomic and plasmid DNA isolation, and stock cultures for long-term storage on silica gel. Two reference strains of *Cmm* (obtained from E. Braun, Iowa State University, Ames, IA), were included as comparisons (Table 1). The morphological characteristics of each of the 37 strains plus the two *Cmm* strains in terms of size (mean colony diameter in mm), color and fluidity were recorded after growth on NBY for 5–7 days at 25°C.

**Table 1:** Morphological and genetic characterization of 37 putative strains of *Clavibacter michiganensis* subsp. *nebraskensis* collected from Goss’s leaf blight symptomatic and asymptomatic maize leaves compared with two strains of *Clavibacter michiganensis* subsp. *michiganensis*.

Strain ID	Field, County, State*	Source^†^	Year	Morphology and color^‡^	Pathogenic	Groups^¶^	PCR-RFLP groups**		Identity^††^	Plasmid
							*rec*A	*rpo*D		
C4	1, Iowa, IA	Epiphytic	2012	Small mucoid, orange	Yes	1	I	I	*Cmn*	-
C5	1, Iowa, IA	Epiphytic	2012	Small mucoid, orange	No	2	III	II	*Cm*	-
C12	1, Iowa, IA	Epiphytic	2012	Small mucoid, orange	Yes	1	I	I	*Cmn*	-
FN	2, Boone, IA	Diseased	2013	Small mucoid, orange	Yes	1	I	I	*Cmn*	-
HF2	3, Story, IA	Epiphytic	2012	Large mucoid, orange	Yes	3	I	I	*Cmn*	-
HI 11–5	4, Story, IA	Epiphytic	2013	Small mucoid, orange	Yes	1	I	I	*Cmn*	-
NE2	5, Grant, NE	Epiphytic	2012	Small mucoid, orange	Yes	1	I	I	*Cmn*	+
CL1	6, Carroll, IA	Diseased	2013	Small mucoid, orange	Yes	1	I	I	*Cmn*	+
C10	1, Iowa, IA	Epiphytic	2012	Large mucoid, orange	No	4	III	II	*Cm*	-
HF4	3, Story, IA	Epiphytic	2012	Large mucoid, orange	No	4	I	I	*Cmn*	-
NE1	5, Grant, NE	Epiphytic	2012	Large mucoid, orange	No	4	III	II	Cm	+
HI 4–5	4, Story, IA	Epiphytic	2013	Large mucoid, orange	Yes	3	I	I	*Cmn*	-
HI 6–5	4, Story, IA	Epiphytic	2013	Small mucoid, orange	Yes	1	I	I	*Cmn*	-
CL4	6, Carroll, IA	Diseased	2013	Large mucoid, orange	Yes	3	I	I	*Cmn*	-
GIL1	7, Story, IA	Diseased	2013	Large mucoid, orange	Yes	3	I	I	*Cmn*	-
GIL3	7, Story, IA	Diseased	2013	Large mucoid, orange	Yes	3	I	I	*Cmn*	-
NE6	5, Grant, NE	Epiphytic	2011	Large fluidal, yellow	No	5	II	na	*Cm*	-
BR2	8, Boone, IA	Epiphytic	2011	Large fluidal, yellow	No	5	II	na	*Cm*	-
BS2	9, Boone, IA	Epiphytic	2011	Large fluidal, yellow	No	5	II	na	*Cm*	-
C8	1, Iowa, IA	Epiphytic	2012	Small mucoid, orange	No	2	IV	na	*Cm*	-
G1	10, Story, IA	Epiphytic	2012	Large fluidal, yellow	No	5	II	na	*Cm*	-
C2	1, Iowa, IA	Epiphytic	2012	Small mucoid, orange	Yes	1	I	I	*Cmn*	-
C3	1, Iowa, IA	Epiphytic	2012	Small mucoid, orange	Yes	1	I	I	*Cmn*	-
C7	1, Iowa, IA	Epiphytic	2012	Large mucoid, orange	Yes	3	I	I	*Cmn*	-
HF1	3, Story, IA	Epiphytic	2012	Large mucoid, orange	Yes	3	I	I	*Cmn*	-
HI 2-AN	4, Story, IA	Epiphytic	2013	Large mucoid, orange	Yes	3	I	I	*Cmn*	-
HI 2-BS	4, Story, IA	Epiphytic	2013	Small mucoid, orange	Yes	1	I	I	*Cmn*	-
HI 2–4	4, Story, IA	Epiphytic	2013	Large mucoid, orange	Yes	3	I	I	*Cmn*	-
HI 11–7	4, Story, IA	Epiphytic	2013	Large mucoid, orange	Yes	3	I	I	*Cmn*	-
HI 11–8	4, Story, IA	Epiphytic	2013	Large mucoid, orange	Yes	3	I	I	*Cmn*	-
HI 6–4	4, Story, IA	Epiphytic	2013	Large mucoid, orange	Yes	3	I	I	*Cmn*	-
HI 7–7	4, Story, IA	Epiphytic	2013	Large mucoid, orange	Yes	3	I	I	*Cmn*	-
NE3	5, Grant, NE	Epiphytic	2012	Small mucoid, orange	Yes	1	I	I	*Cmn*	-
NE4	5, Grant, NE	Epiphytic	2012	Small mucoid, orange	Yes	1	I	I	*Cmn*	-
CL2	6, Carroll, IA	Diseased	2013	Small mucoid, orange	Yes	1	I	I	*Cmn*	-
CL3	6, Carroll, IA	Diseased	2013	Large mucoid, orange	Yes	3	I	I	*Cmn*	-
GIL2	7, Story, IA	Diseased	2013	Large mucoid, orange	Yes	3	I	I	*Cmn*	-
BR-4 (Cmm^‡‡^)	-			Large mucoid, yellow	No	6	nr	III	*Cmm*	+
DR-60 (Cmm)	-			Large mucoid, yellow	No	6	V	III	*Cmm*	+

Pathogenicity tests were done on corn plants that were inoculated with each isolate at the V3–V4 crop developmental stage. Foliar blight severity was rated as the proportion of leaf area affected six days after inoculation.

* GPS co-ordinates of nearest town to which fields were located are provided in S1 Table.

^**†**^ Epiphytic, strain recovered from asymptomatic maize leaves; diseased, strain recovered from maize leaves with symptoms of Goss’s leaf blight

**Fig 1:**
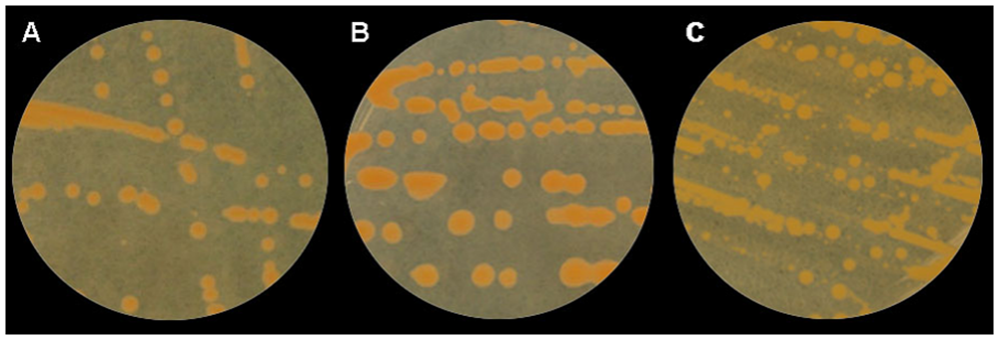
Examples of colony morphology after 5 days growth on nutrient broth yeast extract (NBY) medium of 37 putative isolates of *Clavibacter michiganensis* subsp. *nebraskensis*. A small mucoid orange colony; B large mucoid orange colony; and C large fluidal yellow colony.

^‡^ Colony size was measured after 5 days growth on CNS medium [12] using a ruler and divided into three groups as A, small mucoid orange, (2–3 mm); B, large mucoid orange (3–4 mm) and C, large fluidal yellow (3–4 mm or larger in diameter) (see Fig 1).

^¶^ Groups based on colony morphology after 5 days’ growth on NBY medium and pathogenicity on maize

** PCR-RFLP of housekeeping genes *rec*A and *rpo*D [24]. Numbers in columns represent fragment patterns; na, not amplified; nr, not restricted.

^††^Strains identified as *Clavibacter michiganensis* subsp. *nebraskensis* based on PCR-RFLP pattern of *rec*A and *rpo*D genes [24], all belonged to Groups 1 and 3; *Cm* stands for *Clavibacter michiganensis*.

^‡‡^ *Clavibacter* michiganensis subsp. *Michiganensis*

### Pathogenicity and comparative aggressiveness of isolates

All 37 putative strains of *Cmn* and the two *Cmm* strains were tested for pathogenicity and aggressiveness on the susceptible maize hybrid DKC55-09 in the greenhouse. The *Cmn* strain 91-R (obtained from C. Block, USDA Plant Introduction Station, Ames, IA), which is a rifampicin-tolerant derivative, was included as a reference for pathogenicity. Inoculum for pathogenicity tests was prepared by flooding 3 day-old cultures of the bacterium on NBY plates with 10 ml of phosphate-buffered saline (PBS) and gently scraping the bacterial cells off the surface of the media. Each bacterial suspension was adjusted to an OD_600nm_ of 0.04 (10^6^ CFUs ml^-1^). Two seeds were sown per pot (25.4 cm in diameter) in a peat moss: metro mix: coarse perlite (4:3:4) and plants were grown to the V3 crop developmental stage before inoculation [20]. The third leaf of each plant was wounded by making a cut across 3 veins on one side of the midrib, half way between the leaf sheath and the tip of the leaf. Ten microliters of the inoculum was dropped on the wound using a 25 μl 702N Hamilton syringe (Hamilton CO. Reno, Nevada). Only the third leaf was inoculated per plant and six plants were inoculated per strain. The experimental setup was a completely randomized design and the plants were maintained at a temperature of 25–30°C with a 14 h photoperiod on a greenhouse bench. Disease severity was defined by the proportion of the leaf area affected, and disease ratings started at 6 days after inoculation (DAI) and thereafter were done on every fourth day for 28 DAI. Throughout the experiments, pots were watered daily and plants were fertilized once a week with a liquid fertilizer (NPK: 15–5–15; Miracle-Gro, The Scotts Co., Marysville, OH) that was supplemented with Ca(NO3)_2_ and MgSO_4_ micronutrients at the rate of 43 g/L and 22 g/L, respectively. This experiment was conducted twice and the bacterial strains were re-isolated from the diseased leaves at the end of each experiment to fulfill Koch’s postulates [21].

The leaf blight disease severity ratings were converted to area under the disease progress curve (AUDPC) using the trapezoidal method [22] and analysis of variance was performed using PROC GLIMMIX of SAS software (version 9.3, SAS Institute, Cary, NC), in which plant and leaf were considered random. Mean comparisons were done using Tukey’s test.

### DNA extraction and plasmid isolation

Genomic DNA of each isolate was extracted from a loopful of 6 day old cultures grown on NBY suspended in 1 ml of PBS using a PowerLyser^™^ UltraClean^®^ Microbial DNA Isolation Kit (MOBIO, Cleveland, CA) following the manufacturer’s instructions. Cultures for the isolation of plasmids were grown overnight in 150 ml of TBY plus glucose media [23] on a 250rpm shaker at 25°C. The cells were then pelleted by centrifugation and the plasmids were isolated using the Qiagen plasmid mini kit (Qiagen Sciences, Germantown, MD) following the manufacturers instructions with a few modifications. The bacterial pellet was resuspended in 10ml of buffer PI containing 1mg/ml of lysozyme and incubated at 37°C for 37 mins. Similarly in subsequent steps, 10 ml of buffers P2 and P3 were used. Plasmid DNA was eluted with 30 μl of TE buffer warmed to 65°C.

### Confirmation of identification with PCR-RFLP using housekeeping genes recA and rpoD

The housekeeping genes *rec*A and *rpo*D were amplified using the following primers: for *rec*A, the forward primer *rec*AF (5’-TCGGCAAGGGCTCGGTCATGC- 3’) and reverse primer *rec*AR (5’-GGTCGCCRTCGTASGTGTACCA- 3’). The forward and reverse primers used for *rpo*D were *rpo*DF (5’-ATGGTGCTGTCGAACAAGGA- 3’) and *rpo*DR (5’-CGATCTGGTCGAGSGTCTT- 3’), respectively [24]. PCR amplification was performed using a TopTaq master mix kit in 50 μl reactions. The DNA concentration was adjusted to 20 ng/ul and amplifications were performed as described by Waleron and colleagues [24] in a BIO RAD T100^™^ thermocycler with the following conditions: an initial denaturation at 95°C for 3 min, 32 cycles of denaturation at 94°C for 1 min, annealing at 62°C, for 1 min for *rec*A, and 58°C or 60°C for 1 min for *rpo*D, extension at 72°C for1min, and a final extension at 72°C for 5min. For most of the PCR reactions an annealing temperature of 62°C and 58°C was used for *rec*A and *rpo*D respectively. Following PCR amplification, the products were restricted with the endonuclease *Bst*UI, which is an isoschizomer of *Fnu*DII [24]. Digestion was carried out in a total volume of 50 μl containing 1 μl of *Bst*UI, 5 μl of 10X NEBuffer, 24 μl of molecular grade water and 20 μl of DNA template. The reaction mixture was incubated for 30 min at 60°C. After digestion, the reaction mixture was concentrated for 20 min at medium heat (43°C) in a DNA concentrator to half the volume. The resultant fragments of DNA were separated in a 12% polyacrylamide gel at 200 V for 5–6 h in 0.5X TBE buffer and visualized with UV light after staining in ethidium bromide (0.5 μg ml^-1^) [24]. The PCR-RFLP using polyacrylamide gel was carried out twice under same conditions.

The protocol of Waleron et al. [24] was later modified in that resolution of the restricted fragments was done in a 4% agarose gel prepared in sodium borate solution [25]. The DNA fragments were electrophoresed at 100 V for 2 h and visualized with UV light after staining in ethidium bromide (0.5 μg ml^-1^). This experiment was done twice.

### Comparative sequence analyses of putative virulence genes

Published virulence factors in the subspecies *sepedonicus* and *michiganensis* [17] were used to search for corresponding homologs in the genome of *Cmn* in order to differenciate between the non-pathogenic and pathogenic strains of *Cmn*. In addition, several serine proteases that encode for toxin-antitoxin systems and the chloride anion channel gene [26, 27] were selected for sequencing. The sequences of these putative virulence factors were identified in the genome of the *Cmn* isolate NCPP 2581 (NCBI Reference Sequence: NC_020891.1, released but not yet published) found on the NCBI nucleotide database (http://www.ncbi.nlm.nih.gov/nuccore/473832060?report=graph). Primer pairs, designed with sequences of genomic loci that flanked each gene using the DNADynamo sequence analysis program (Blue tractor software Ltd, UK), were used to amplify the complete sequences of the target genes. The PCR products were sequenced at the DNA facility of Iowa State University. Sequences were aligned and manually edited using the biological editor, BioEdit [28] before they were translated and aligned using the DNADynamo sequence analysis program.

## Results

### Morphological characteristics of isolates, pathogenicity, and aggressiveness on maize

The 37 putative strains of *Cmn* were classified into six groups based on colony morphology after 5 days’ growth on NBY medium (Fig 1) and pathogenicity on maize (Table 1). Thirteen strains that produced small, mucoid orange colonies and were pathogenic on maize, were classified into Group 1. Ten of these strains were recovered from asymptomatic maize leaves and the remaining three from Goss’s leaf blight lesions. Group 2 contained two epiphytic strains that were morphologically similar to strains from Group 1 but were not pathogenic on maize. Group 3 contained strains recovered from asymptomatic (10 strains) and symptomatic maize leaves (5 strains) that produced large, mucoid, orange colonies and were all pathogenic on maize. Three epiphytic strains also produced large, mucoid orange colonies but did not cause disease symptoms on maize and were classified as Group 4. Four strains, that produced large fluidal yellow colonies and were not pathogenic on maize, were classified as Group 5. All four strains were recovered from asymptomatic leaves. The two *Cmm* reference strains included in this study produced large, mucoid yellow colonies on NBY and were non-pathogenic on maize (Group 6). Thus, based on colony morphology (orange mucoid) and pathogenicity on maize, a total of 28 strains (20 epiphytic and 8 from diseased maize leaves) were identified as *Cmn*.

**Fig 2:**
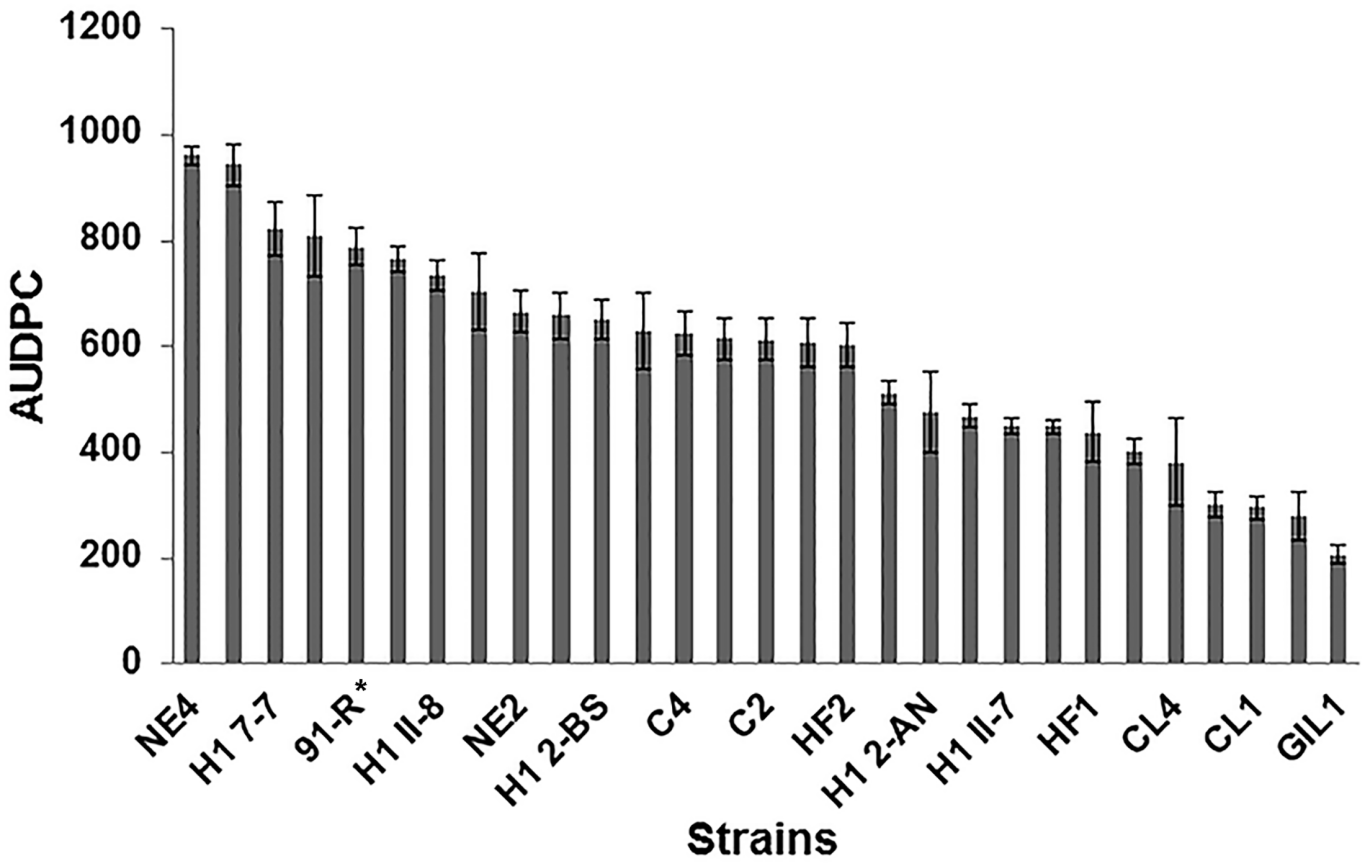
Mean aggressiveness (n = 12 plants) of 28 *Clavibacter michiganensis* subsp. *nebraskensis* strains estimated from foliar blight severity on maize hybrid DKC 55–09. Plants were inoculated at the V3–V4 crop developmental stage and foliar blight severity was rated as the proportion of leaf area affected six days after inoculation. Bars represent standard errors of the mean. *Strain 91-R was included as a reference for pathogenicity in this experiment.

Analysis of variance showed significant differences in aggressiveness among the 28 *Cmn* strains (*P* < 0.0001). Disease severity (proportion of the leaf area affected) ranged from 16.0 to 78%. Strain NE4 was the most aggressive while GIL 1 was the least aggressive on the maize hybrid DKC55-09 (Fig 2).

Among the 37 putative *Cmn* strains, only NE1, NE2 and CL1 contained single large plasmids that were estimated to be 70 kb (Table 1).

### Identification of *Cmn* using PCR-RFLP analysis

**Fig 3:**
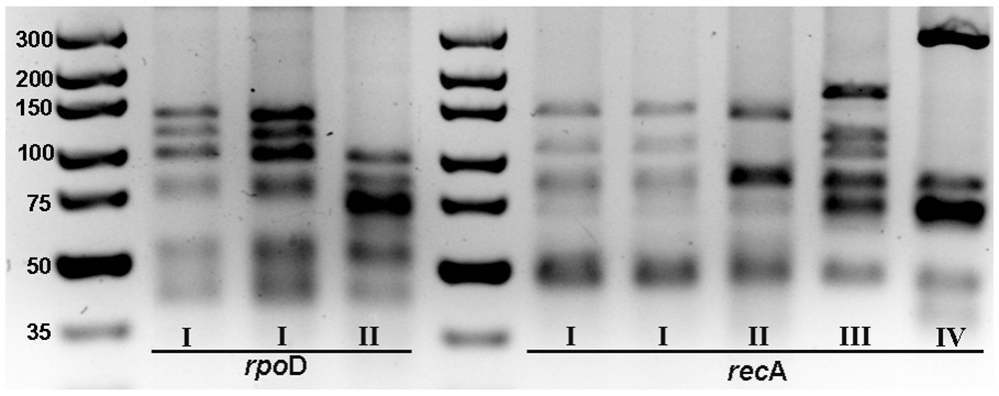
Representative RFLP patterns obtained after restriction of rpoD and recA with BstU1 for 37 putative isolates of *Clavibacter michiganensis* subsp. *nebraskensis* and two strains of *Clavibacter michiganensis* subsp. *michiganensis*. Roman numerals correspond to pattern types observed. Molecular size marker: GeneRuler 100 bp DNA Ladder.

The *rpo*D and *rec*A genes were successfully amplified from all 37 putative *Cmn* strains and the size of each amplicon was consistent with that reported by Waleron et al. [24]. Digestion of the *rpo*D and *rec*A amplicons using *Bst*U1 resulted in two and four restriction patterns, respectively (Table 1, Fig 3). When the amplified *rec*A fragment was digested with *Bst*U1, RFLP pattern I was observed for all 27 strains that were pathogenic as well as strain HF4, which was not pathogenic on maize. Six bands of size ranging from 50 to 140 bp were observed on the polyacrylamide gel while only five bands of the same size range were observed with 4% agarose gel. RFLP pattern I was similar to PCR-RFLP pattern 3 reported by Waleron et al. [24] for *Cmn*. Three additional RFLP patterns, II, III and IV, were observed for strains representative of Groups 2, 4 and 5 respectively, that were not pathogenic on maize. The RFLP pattern observed with *Bst*U1digestion of *rec*A amplicon of *Cmm* strain DR-60 was consistent with that reported by Waleron et al. [24]. The *rec*A amplicon of *Cmm* strain BR-4 could not be digested with the enzyme (data not shown).

For *Bst*U1digestion of *rpo*D fragment, two RFLP patterns, I and II, were observed for all the 37 putative *Cmn* strains. RFLP pattern I that had six bands (40 to 140 bp) on both polyacrylamide and agarose gels was observed for all strains that were pathogenic on maize, as well as HF4 (Table 1; Fig 3). The first three bands of the pattern on both gels were larger (approximately 40, 30 and 20 bp for the first, second and third band, respectively) than those reported by Waleron et al. [24], while the smaller bands were smaller than reported. Pattern II was obtained for three non-pathogenic strains (Table 1). The RFLP patterns observed for *Bst*U1digestion of *rpo*D for the two strains of *Cmm* that were included in the study were consistent with those reported by Waleron et al. [24].

### Comparative sequence analyses of putative virulence genes

Thirty-three genes that encode for putative virulence factors identified in the *Cmn* genome database were successfully amplified and sequenced for *Cmn* strain GIL1 (pathogenic) and *Cmn* strain HF4 (non-pathogenic on maize). The pathogenic strain GIL1 had identical gene sequences with the reference *Cmn* strain NCPPB 2581. When the GIL1 gene sequences were compared to those of HF4, polymorphisms were detected in the Cellulase A, two endoglucanase genes, the xylanase B gene, and the pectate lyase gene (Table 2; S1 File). The cellulase A gene in strain HF4 was 1100 base pairs long with a 18 nucleotide insertion that was absent in strain GIL1. An in silico translation of the nucleotide sequence revealed the 18 nucleotide insertion was a repeat that corresponded to the amino acid sequence “PTPPSQ” (S2 File). This amino acid sequence was repeated three times in the reference strain NCPPB 2581 and GIL1, while HF4 contained an additional repeat of the sequence bringing the total to four. One of the endoglucanase genes (*Cmn* 2651) was 1386 nucleotides in length with a 66 nucleotide insertion located between nucleotides 419–486 in strain HF4 that was absent in strain GIL1. An in silico translation of the gene sequence showed the insertion corresponded to 22 additional amino acids in which no repeats could be identified (S2 File). The second endoglucanase (*Cmn* 2650), a xylanase B gene and a pectate lyase gene (*Cmn* 2654) differentiated the two strains by 3, 6 and 1 single nucleotide polymorphisms (SNPs) respectively. Only one of the SNPs in *Cmn* 2650 resulted in a change in amino acid sequence from A to G. Four of the SNPs in the Xylanase B gene resulted in the amino acids A, D, P and L instead of S, Y, L, and V respectively in the reference strain and the rest were silent mutations (S2 File).

**Table 2:** Nucleotide polymorphisms in the sequences of putative virulence factors found in the genome of *Clavibacter michiganensis* subsp. *nebraskensis* strains GIL1 and HF4. PCR primers flanking each locus were used in sequencing reactions to obtain the complete sequence of each gene.

Locus in *Cmn* genome (Isolate NCPPB 2581)	Putative function	Nucleotide differences
*Cmn* 00734	Chloride anion channel	No difference
*Cmn* 00144	Secreted cellulase A	Indel (18 nucleotides)
*Cmn* 02650	Endoglucanase	3 SNPs
*Cmn* 02651	Endoglucanase	Indel (66 nucleotides)
*Cmn* 00792	Translocase glycosyl hydrolase	No difference
*Cmn* 01115	Polysaccharide deacetylase	No difference
Xys A	Endo-1, 4-beta xylanase A	No difference
Xys B	Endo-1, 4-beta xylanase B	6 SNPs
Pga A	Polygalacturonase A	No difference
*Cmn* 01173	Secreted serine peptidase	No difference
*Cmn* ppaF	Secreted serine peptidase	No difference
*Cmn* 457	Secreted serine peptidase	No difference
*Cmn* sbtC	Serine peptidase	No difference
*Cmn* sbtB	Serine peptidase	No difference
*Cmn* 2417	Secreted serine peptidase	No difference
*Cmn* 2381	Secreted serine peptidase	No difference
*Cmn* 2235	Secreted serine peptidase	No difference
*Cmn* 1337	Secreted serine peptidase	No difference
*Cmn* 1248	Secreted serine peptidase	No difference
*Cmn* 00106	Toxin-antitoxin system	No difference
*Cmn* 00626	Toxin-antitoxin system	No difference
*Cmn* 00771	Toxin-antitoxin system	No difference
*Cmn* 01077	Toxin-antitoxin system	No difference
*Cmn* 01078	Toxin-antitoxin system	No difference
*Cmn* 02136	RTX toxin	No difference
*Cmn* 02626	Toxin component	No difference
*Cmn* 02669	Toxin component	No difference
*Cmn* 02707	Toxin gene	No difference
*Cmn* 00414	Protein kinase	No difference
*Cmn* 0118	Exported toxin	No difference
*Cmn* 02101	TetR lipase/esterase	No difference
*Cmn* 00283	Glycosyl transferase	No difference
*Cmn* 02654	Pectate lyase	I SNP

Sequences were aligned and examined for nucleotide sequence differences. Primers were designed from reference strain NCPPB 2581 genomic sequence.

To further characterize the nucleotide differences we observed in the cellulose A and endogluconase (*Cmn* 2651) genes, we sequenced each loci in five additional *Cmn* strains (FN, C4, NE3, GIL3, and 91-R) and a non-pathogenic *Clavibacter michiganensis* strain (NE1). The same indel in the cellulase A gene sequence was found. The avirulent strain NE1 and the virulent strains NE3, FN, GIL3, and 91-R were similar and lacked the 18 nucleotide insertion, while the virulent strains C4 and the avirulent strain HF4 contained the 18 nucleotide insertion. Similarly, the sequence of the endoglucanase gene (*Cmn* 2651) in the virulent strains C4, GIL3, and NE3 and the avirulent strain NE1 contained the 66 nucleotide insertion, while the sequence in the two virulent strains FN and 91-R was the same as in the avirulent strain HF4.

## Discussion

In this study we compared the morphology and aggressiveness of 37 strains of putative *Cmn* recovered from maize leaves with Goss’s leaf blight symptoms or asymptomatic leaves from maize plants grown in fields with a history of Goss’s wilt and leaf blight in Iowa and Nebraska. The bacterium was always recovered from symptomatic tissues but the recovery rate of *Cmn* from asymptomatic tissues was lower (approximately 0 to 50 percent). This may have been a function of our sampling technique. There are few data regarding epiphytic colonization of the plant canopy, or individual leaves, by *Cmn*. Such data would enable more targeted sample collection rather than our sampling method that consisted of collecting arbitrary leaves from various positions within the canopy of numerous plants within a field with a history of the disease. We identified 28 of the strains as *Cmn* based on colony morphology, pathogenicity on maize, and PCR-RFLP of housekeeping genes. Of these 28 strains, 20 had been recovered from the phyllosphere of maize leaves.

We found no relationship between colony morphology of putative *Cmn* strains and pathogenicity on maize confirming that it is difficult to identify *Cmn* purely on colony morphology on CNS [15, 29]. In environments where it is important to verify the organism, for example seed laboratories where testing maize seed for which quarantine restrictions have been placed to prevent the introduction of *Cmn*, this is obviously a concern. Consequently, additional tests that usually include pathogenicity assays on maize in the greenhouse are always required to confirm if a putative *Cmn* colony is the pathogen of interest.

We found the PCR-RFLP method described by Waleron et al. [24] was able to correctly identify putative *Cmn* colonies within a couple of days compared to greenhouse pathogenicity tests that take several weeks. This suggests that this method could be useful for diagnosis of *Cmn*. However, there are limitations with the method that should be addressed. Strain HF4 was identified as *Cmn* using this assay, although no symptoms developed when it was inoculated onto maize seedlings indicating it was non-pathogenic. The occurrence of non-pathogenic variants within subspecies of *C*. *michiganensis* is of great concern especially for hosts that are of phytosanitary importance. Detection of non-pathogenic *Cmn* in corn seed that may pose minimal risk to the importing country could result in exclusion of the seed. The existence of non-pathogenic variants in the subspecies *michiganensis* has also been very problematic in the design of assays for the detection and quantification of the pathogen in tomato seed [30]. Louws et al., [31] were unable to distinguish between virulent and avirulent *C*. *michiganensis* subsp. *michiganensis* strains using rep-PCR. When box-PCR and AFLP primers were used to genotype *Cmn* strains, pathogenic and non-pathogenic variants of *Cmn* were indistinguishable [32]. These results are an indication of how closely related the pathogenic and non-pathogenic strains of *Cmn* are and the differences may lie in virulence genes or promoters.

Modification of the PCR-RFLP method in which a 4% agarose gel prepared in sodium borate solution was used rather than a polyacrylamide gel to visualize the digested fragments, makes this *C*. *michiganensis* identification method user-friendly since agarose is easier to use, cheaper and less toxic than acrylamide. However, resolution of bands that differ in size by a few base pairs can be difficult on an agarose gel [33]. This was evident in our study where the restriction pattern we observed for *rec*A had five bands rather than six as was observed on the polyacrylamide gel. Thus on the agarose gel we were unable to resolve the third and fourth bands that Waleron et al. [24] reported. Nevertheless this method could be useful to identify subspecies of *C*. *michiganensis* from environmental samples.

It is unclear why HF4 was non-pathogenic on maize despite it being so similar morphologically and genetically to other *Cmn* strains in our study. Although the presence of plasmids has been associated with virulence in *Cmm* and *Cms*, this is not the case for *Cmn* [19, 34]. Earlier studies have shown that there were no differences in pathogenicity between *Cmn* strains that contained plasmids and those without plasmids [2,13]. In the present study, strains NE2 and CL1 were both pathogenic while NE1 was non-pathogenic, but all three contained plasmids, while the rest of the pathogenic strains were devoid of plasmids. Although these plasmids are yet to be characterized, our data further confirm that *Cmn* virulence factors are not plasmid-borne.

Toxicity genes have been hypothesized to play a role in virulence of *Cmn*, but only one of twelve putative toxin genes in the genome of *Cmn* has been partially characterized. This membrane-active component called the chloride anion channel (CAC) protein was shown to form anion channels in planar lipid bilayers in vitro. The activity of this protein was shown to be similar to that of both colicins and the *Hm*-T toxins. However, its direct role in pathogenicity on maize was not demonstrated [26, 27, 35,36]. In an effort to understand why HF4 was non-pathogenic on maize, we sequenced 33 genes that encode putative virulence factors, including the CAC gene, and compared the sequences to those of other pathogenic strains, and the reference strain NCPP2581 whose genome has been sequenced. Differences in sequence data were detected only in 5 of the 33 genes, but the sequence polymorphisms observed were unrelated to pathogenicity. Moreover, we found no sequence polymorphism within the CAC gene in both pathogenic and non-pathogenic strains. Similarly, there was no relationship between sequence polymorphisms observed in four putative virulence genes and the ability of the strains to be pathogenic on maize. However, these data do not eliminate the role of CAC or the other putative virulence factors in the disease causing process. Gene regulation was not tested in this study. Thus it is possible that differences at the level of regulation of gene expression may be responsible for virulence. These data further confirm reports that pathogenicity determinants in *Cmn* may be different from those in its close relatives, *Cmm* and *Cms* [17]. Currently in a collaborative project, the genomic sequence of strain HF4 has been completed and is being annotated. Comparative genomics and subsequent functional analysis should enable the virulence factors in *Cmn* to be identified.

## Supporting Information

S1 File Multiple sequence alignment of the ORFs of Cellulase A (Cmn00144), endoglucanase (Cmn 02650 and Cmn 02651), the xylanase B gene, and pectate lyase (Cmn02654) of *Clavibacter michiganensis* subsp. *michiganensis* strains NE3 and HF4. (PDF)

S2 File In silico translation of the Cellulase A (Cmn00144), endoglucanase (Cmn02650 and Cmn02651), the xylanase B gene, and the pectate lyase gene (Cmn02654) of *Clavibacter michiganensis* subsp. *michiganensis* strains NE3 and HF4. (PDF)

S1 Table GPS coordinates of nearest town to which fields are located. (DOCX)
