# Supplementary material for: Characterization and Comparison of Clavibacter michiganensis subsp. nebraskensis Strains Recovered from Epiphytic and Symptomatic Infections of Maize in Iowa
Source: PLoS One. 2015 Nov 24;10(11):e0143553. doi: 10.1371/journal.pone.0143553 (PMC4658045; doi:10.1371/journal.pone.0143553)
Supplement: S1 File — (PDF) [file pone.0143553.s001.pdf]

```
Cmn00144_CEL_A_GENE
NE3 TCAGTGCACCGGATAGAACCGGCGCGCCGTACTCTGGATCTCCCCTGGTG 50
HF4 TCAGTGCACCGGATAGAACCGGCGCGCCGTACTCTGGATCTCCCCTGGTG 50

NE3 CCATTCGGATTCCGGGGAGAACGACCGAGCGACCGTATTGGTCAGTCACT 100
HF4 CCATTCGGATTCCGGGGAGAACGACCGAGCGACCGTATTGGTCAGTCACT 100

NE3 CTAACGGTCATCGGGCCGTCCGGGATAGGTGAAGGTGTCACCCAGTACCC 150
HF4 CTAACGGTCATCGGGCCGTCCGGGATAGGTGAAGGTGTCACCCAGTACCC 150

NE3 GTAGTCAGTGCGGCTCAGGCCCAGCCACTGTCCATCGGCTTGGACTTCAA 200
HF4 GTAGTCAGTGCGGCTCAGGCCCAGCCACTGTCCATCGGCTTGGACTTCAA 200

NE3 CATGGTCGATGGGGTTGCCGGCGTTCAGAATCTGCAGACCGGCCCACCAT 250
HF4 CATGGTCGATGGGGTTGCCGGCGTTCAGAATCTGCAGACCGGCCCACCAT 250

NE3 CGGGATGAGCCCTCCTTGACCCGGACGGCGACATCGGGCACGTCCGGATC 300
HF4 CGGGATGAGCCCTCCTTGACCCGGACGGCGACATCGGGCACGTCCGGATC 300

NE3 CCGGACGGTGGTGTAGCTGATCGGGATGACGCCGGCATCGAAGTCGCCGA 350
HF4 CCGGACGGTGGTGTAGCTGATCGGGATGACGCCGGCATCGAAGTCGCCGA 350

NE3 CGGCACGGAACGCTTCCTCGCTCAAGTCGAGATGTCCGATCTCGCACTCG 400
HF4 CGGCACGGAACGCTTCCTCGCTCAAGTCGAGATGTCCGATCTCGCACTCG 400

NE3 TGGCATTGGTCGACGATTTGGACGCGGACGGTGCCCTTGGGGCCCGTGAC 450
HF4 TGGCATTGGTCGACGATTTGGACGCGGACGGTGCCCTTGGGGCCCGTGAC 450

NE3 GAGGAGGTACGAACCGCACGCGGCGGCTCCGCTGTACTCGGGGCTGCTGA 500
HF4 GAGGAGGTACGAACCGCACGCGGCGGCTCCGCTGTACTCGGGGCTGCTGA 500

NE3 CCGCAACGTACATGCGGTCGGCGGGGACAGCCGGCATGGAGCAGTTGCCA 550
HF4 CCGCAACGTACATGCGGTCGGCGGGGACAGCCGGCATGGAGCAGTTGCCA 550

NE3 TTCGCGATCGTGTTGCCGGTACCGAGCGAGTAGTGCGTGGCACGGCCGTG 600
HF4 TTCGCGATCGTGTTGCCGGTACCGAGCGAGTAGTGCGTGGCACGGCCGTG 600

NE3 CGTCGCCGACTGGGACGGCGGCGTGGGCTGGGACGGCGGCGTGGGCTGGG 650
HF4 CGTCGCCGACTGGGACGGCGGCGTGGGCTGGGACGGCGGCGTGGGCTGGG 650

NE3 ACGGCGGCGTGGGCTGGGACGGCGGCGT------------------TCCG 682
HF4 ACGGCGGCGTGGGCTGGGACGGCGGCGTGGGCTGGGACGGCGGCGTTCCG 700
                                ******************
NE3 GCCGCTGTGGCACTGATTTGAGGCGAAGAGGGAGCGGGTCCACCCGCCAA 732
HF4 GCCGCTGTGGCACTGATTTGAGGCGAAGAGGGAGCGGGTCCACCCGCCAA 750

NE3 CTGCACGCCGACGCTAACGGTCTGTCCGGCGGCGAGCTCGCTTGCCCAGT 782
```

HF4 CTGCACGCCGACGCTAACGGTCTGTCCGGCGGCGAGCTCGCTTGCCCAGT 800

NE3 CCGTGCCGGTGCAGGTCACGGTGTTGGAGGCGACGCTACAGCGCATGCCC 832
HF4 CCGTGCCGGTGCAGGTCACGGTGTTGGAGGCGACGCTACAGCGCATGCCC 850

NE3 CAACTGTTGACGACGCCGGTGGTTTCCGGGCTGGCCCATGAGACGGTCCA 882
HF4 CAACTGTTGACGACGCCGGTGGTTTCCGGGCTGGCCCATGAGACGGTCCA 900

NE3 TCCCGTGACAGCGTCTGTCGCGGTGACGTCGAGGTTCGCCACGTAGCCGG 932
HF4 TCCCGTGACAGCGTCTGTCGCGGTGACGTCGAGGTTCGCCACGTAGCCGG 950

NE3 ATGCCCAGGACCCGCCCGGCTGCCACTTCGCTGAAATCGCGCCTGGCTTC 982
HF4 ATGCCCAGGACCCGCCCGGCTGCCACTTCGCTGAAATCGCGCCTGGCTTC 1000

NE3 GCCGGACCTGGCTGCGATTCGGATCCGCCAGATCCCGGACCCGCTGCGGT 1032
HF4 GCCGGACCTGGCTGCGATTCGGATCCGCCAGATCCCGGACCCGCTGCGGT 1050

NE3 GGCGGCTGAGGCAGGCGCGGCGAATGCGCCAAACGTGGCACCGACTGCCA 1082
HF4 GGCGGCTGAGGCAGGCGCGGCGAATGCGCCAAACGTGGCACCGACTGCCA 1100

NE3 T 1083
HF4 T 1101

Cmn02650_Endoglucanase_gene

```
GIL1 ATGCAGCACCACCGTCACCCCATCCGCACCGCGACCACCCTCGTCCTGGG 50
HF4  ATGCAGCACCACCGTCACCCCATCCGCACCGCGACCACCCTCGTCCTGGG 50

GIL1 TCTCGCCCTCACCGCCGGGATCCTCACCGCAGCATCCCCCGCCAGCGCCG 100
HF4  TCTCGCCCTCACCGCCGGGATCCTCACCGCAGCATCCCCCGCCAGCGCCG 100

GIL1 CCACCACCGGCCCGGCCCCGACCGTCGCGAGCGGCGCTCCGACCGTCTCC 150
HF4  CCACCACCGGCCCGGCTCCGACCGTCGCGAGCGCCGCTCCGACCGTCTCC 150
                     *                *
GIL1 GACGCGCGGCTCCGCTTCGGCGTCGCCACCCCCGGCGGCCCGACCGACAC 200
HF4  GACGCGCGGCTCCGCTTCGGCGTCGCCACCCCCGGCGGCCCGACCGACAC 200

GIL1 CGGCGAGCTCGACGCCGTAGCCCAGCAGGTCGGCGAGGACCCGAGCATCG 250
HF4  CGGCGAGCTCGACGCCGTAGCCCAGCAGGTCGGCGAGGACCCGAGCATCG 250

GIL1 TGCTCGCGTACGCCGACTTCACGCAGGCGCCGCCCATCGCGGCCCTCGAC 300
HF4  TGCTCGCGTACGCCGACTTCACGCAGGCGCCGCCCATCGCGGCCCTCGAC 300

GIL1 TCGGTCGCGGCGCGCGGTGCCGAGACGCTCCTCACGTGGGAGCCGTGGAC 350
HF4  TCGGTCGCGGCGCGCGGTGCCGAGACGCTCCTCACGTGGGAGCCGTGGAC 350

GIL1 GGCCGGCGCCGGCGTGGATCAGCCCACGTTCACGAACGCGAGCATCGCCG 400
HF4  GGCCGGCGCCGGCGTGGATCAGCCCACGTTCACGAACGCGAGCATCGCCG 400

GIL1 CGGGCGACCACGACGCCTACATCCGCGAGTGGGGCGCCGCCCTGGCGAAG 450
HF4  CGGGCGACCACGACGCCTACATCCGCGAGTGGGGCGCCGCCCTGGCGAAG 450

GIL1 TGGGGCGGACCCGTCTCCCTCCGCTACGCGCACGAGATGAACGGCGACTG 500
HF4  TGGGGCGGACCCGTCTCCCTCCGCTACGCGCACGAGATGAACGGCGACTG 500

GIL1 GTACCCGTGGGCCGACGGCGTCAACGGCAACGCGCCGGGCTCCTACGCCG 550
HF4  GTACCCGTGGGCCGACGGCGTCAACGGCAACGCGCCGGGCTCCTACGCCG 550

GIL1 CCGCATTCCGGCACGTGCACGACGTCGTCGTGGGCGCCGGCGCGACCAAC 600
HF4  CCGCATTCCGGCACGTGCACGACGTCGTCGTGGGCGCCGGCGCGACCAAC 600

GIL1 GTGCGCTGGGTGTGGACGCCGAACGTGCCGTACACCGGATCCACCGCCCT 650
HF4  GTGCGCTGGGTGTGGACGCCGAACGTGCCGTACACCGGATCCACCGCCCT 650

GIL1 CGCGGGGCTCTACCCGGGCGCCGGCTACGTCGACGTCGTCGGCCTCGACG 700
HF4  CGCGGGGCTCTACCCGGGCGCCGGCTACGTCGACGTCGTCGGCCTCGACG 700

GIL1 GCTACAACTGGGGCTCCGTCGCCGGCCAGAGCTGGACCGCGCCGAGCGAC 750
HF4  GCTACAACTGGGGCTCCGTCGCCGGCCAGAGCTGGACCGCGCCGAGCGAC 750

GIL1 CTATTCGGCGCCGGTCTCGAGCAGCTGCGCGCCATCGCGCCGGGGAAGCC 800
HF4  CTGTTCGGCGCCGGTCTCGAGCAGCTGCGCGCCATCGCGCCGGGGAAGCC 800
```

```
       *

GIL1 GATCGTCATCGCGGAGACCGCATCCTCCGAGGTCGGCGGATCCAAGGCCC 850
HF4  GATCGTCATCGCGGAGACCGCATCCTCCGAGGTCGGCGGATCCAAGGCCC 850

GIL1 AGTGGGACGGCGAGCTCGTCTCCTTCCTGCAGGCCCAGACCGACGTCGTC 900
HF4  AGTGGGACGGCGAGCTCGTCTCCTTCCTGCAGGCCCAGACCGACGTCGTC 900

GIL1 GCCTTCGTCTGGTTCGACATGGACAAGGAGGCGGACTGGCGCATCGGCAG 950
HF4  GCCTTCGTCTGGTTCGACATGGACAAGGAGGCGGACTGGCGCATCGGCAG 950

GIL1 CTCCGCGTCGTCCGCCACCGCCATCCACGACGCGCTCGCCGCCCGTCGCC 1000
HF4  CTCCGCGTCGTCCGCCACCGCCATCCACGACGCGCTCGCCGCCCGTCGCC 1000

GIL1 TCCAGTAG 1008
HF4  TCCAGTAG 1008
```

Cmn02651_Endoglucanase_gene

```
NE3 ATGCCCACTCGCCGCCGTCTCAAATGCGCCGTCGTCACCGCCGTCGCACC 50
HF4 ATGCCCACTCGCCGCCGTCTCAAATGCGCCGTCGTCACCGCCGTCGCACC 50

NE3 GGCAGTCGTGACAGCCACCGTGCTCGTCGCTCCCGCGGCCGGCGCCCGCG 100
HF4 GGCAGTCGTGACAGCCACCGTGCTCGTCGCTCCCGCGGCCGGCGCCCGCG 100

NE3 CCGCCTCGGCGCAGGCCGCACCCACGGCCCCCGCCGCGACCGTCCTCTCC 150
HF4 CCGCCTCGGCGCAGGCCGCACCCACGGCCCCCGCCGCGACCGTCCTCTCC 150

NE3 GTCGCCTCCGGCCCGGTCGGCACGCCCGTCACCGTCACCGGCACGGGCTT 200
HF4 GTCGCCTCCGGCCCGGTCGGCACGCCCGTCACCGTCACCGGCACGGGCTT 200

NE3 CCCCGCGAAGAAGGCCGCGGTCGTCGCCGTCGGTTCGACCACGAAGCGCA 250
HF4 CCCCGCGAAGAAGGCCGCGGTCGTCGCCGTCGGTTCGACCACGAAGCGCA 250

NE3 TCACGACCACCGCCACGGGCCGCTTCACGACCGCGATCACCATCCCCCGC 300
HF4 TCACGACCACCGCCACGGGCCGCTTCACGACCGCGATCACCATCCCCCGC 300

NE3 ACCTCGCTCTCCACCATCCGGATCACCGCGACGGCCGGCACCCGCTCGGC 350
HF4 ACCTCGCTCTCCACCATCCGGATCACCGCGACGGCCGGCACCCGCTCGGC 350

NE3 CGGGGCCCCCTTCACCGTCACCACGGCCAAGTCGAACGGATCCGCCCGCC 400
HF4 CGGGGCCCCCTTCACCGTCACCACGGCCAAGTCGAACGGATCCGCCCGCC 400

NE3 TGGGCGCGTCCGCCCCGGCCCCCACCACGACCCCGGCGCCCGCTCCTACG 450
HF4 TGGGCGCGTCCGCCCCGGC------------------------------- 419
                       *******************************

NE3 GGCGCGCCCACCTCCACCGCCGCTCCCGCCCCGGCGACCTCCTCGCCCGC 500
HF4 -----------------------------------GACCTCCTCGCCCGC 434
    ***********************************

NE3 GCCCGCCGCATCCGCCGCCCCCGCCATCAGCTCCGCCCGCCTGCGCCTCG 550
HF4 GCCCGCCGCATCCGCCGCCCCCGCCATCAGCTCCGCCCGCCTGCGCCTCG 484

NE3 GCCTCTCGACCCCCGGCGGGCCGACGGCGAACGGCGAGCTCGACGCGGCG 600
HF4 GCCTCTCGACCCCCGGCGGGCCGACGGCGAACGGCGAGCTCGACGCGGCG 534

NE3 TCGACGACGCTCGGCGAGAGCCCCTCCATCGTGATGAGCCACGTCGACTT 650
HF4 TCGACGACGCTCGGCGAGAGCCCCTCCATCGTGATGAGCCACGTCGACTT 584

NE3 CACCCACCCGGCGCCCATCGCCGGGCTCCGGAGCGTGGCCGCGCGCGGCG 700
HF4 CACCCACCCGGCGCCCATCGCCGGGCTCCGGAGCGTGGCCGCGCGCGGCG 634

NE3 CCGACAGCCTCATCACGTGGGAGCCGTGGCAGGGCGGCGCGGGCGTCGAC 750
HF4 CCGACAGCCTCATCACGTGGGAGCCGTGGCAGGGCGGCGCGGGCGTCGAC 684
```

NE3 CAGCCCGCCTACGCGAACGCGCGCATCGTCGCCGGCGACTACGACTCGTA 800
HF4 CAGCCCGCCTACGCGAACGCGCGCATCGTCGCCGGCGACTACGACTCGTA 734

NE3 CATCCGCTCGTGGGGCGCCGACCTCGCGAAGTACGGCCAGCCCGTCTACC 850
HF4 CATCCGCTCGTGGGGCGCCGACCTCGCGAAGTACGGCCAGCCCGTCTACC 784

NE3 TGCGCTTCGGCCACGAGATGAACGGCAACTGGTACCCGTGGTCCGACGGC 900
HF4 TGCGCTTCGGCCACGAGATGAACGGCAACTGGTACCCGTGGTCCGACGGC 834

NE3 GTCAACGGCAACGCGTCCGGCTCCTACGTGCAGGCGTGGAAGCACGTGCA 950
HF4 GTCAACGGCAACGCGTCCGGCTCCTACGTGCAGGCGTGGAAGCACGTGCA 884

NE3 CGACCTCGTCGCCGCGCAGGGGGCCACGAACGTGAAGTGGGTGTGGAGCC 1000
HF4 CGACCTCGTCGCCGCGCAGGGGGCCACGAACGTGAAGTGGGTGTGGAGCC 934

NE3 CGAACGTGCCGTACCCGGGATCCACCGACCTCGCCTCCCTCTACCCCGGC 1050
HF4 CGAACGTGCCGTACCCGGGATCCACCGACCTCGCCTCCCTCTACCCCGGC 984

NE3 GCCGACCAGGTCGACGTCGTCGCGCTCGACGGCTACAACTGGGGCGCCGT 1100
HF4 GCCGACCAGGTCGACGTCGTCGCGCTCGACGGCTACAACTGGGGCGCCGT 1034

NE3 GCCCGGCCAGCGCTGGACCGCGCCCGCCGACCTCTTCGGCCCCGGAATGG 1150
HF4 GCCCGGCCAGCGCTGGACCGCGCCCGCCGACCTCTTCGGCCCCGGAATGG 1084

NE3 CGCAGCTGCGCGCCGTCGCACCCGGGAAGCCGCTCATCATCGGCGAGGTG 1200
HF4 CGCAGCTGCGCGCCGTCGCACCCGGGAAGCCGCTCATCATCGGCGAGGTG 1134

NE3 GCGTCGGGCGAGACCGGCGGATCCAAGGCCGACTGGGACCGCGACCTCGT 1250
HF4 GCGTCGGGCGAGACCGGCGGATCCAAGGCCGACTGGGACCGCGACCTCGT 1184

NE3 CGCGTACCTCCAGTCGCAGCCCGACGTGCTCGGCTTCGTGTGGTTCGACT 1300
HF4 CGCGTACCTCCAGTCGCAGCCCGACGTGCTCGGCTTCGTGTGGTTCGACT 1234

NE3 TCAAGAAGGAGGAGGACTGGCGGATCGACAGCTCGGCCGCCTCCGCCACC 1350
HF4 TCAAGAAGGAGGAGGACTGGCGGATCGACAGCTCGGCCGCCTCCGCCACC 1284

NE3 GCCCTCCGCGACGCGCTCGCCCTGCGGCGCGGCTGA 1386
HF4 GCCCTCCGCGACGCGCTCGCCCTGCGGCGCGGCTGA 1320

```
XylanaseB_GENE
GIL1 ATGAGCATTCCCCGCACCGACACGTCCGCGCTCGCGGACGTCAGCCCCCC 50
HF4  ATGAGCATTCCCCGCACCGACACGTCCGCGCTCGCGGACGTCAGCCCCCC 50

GIL1 GCGCTTGGGCCGCAGCAGGTTCGCGCCCCGCCCGCGACGCGACCGCCGCC 100
HF4  GCGCTTGGGCCGCAGCAGGTTCGCGCCCCGCCCGCGACGCGACCGCCGCC 100

GIL1 GCGGCCTCGCCGCCCTCACGGCCCTCGGCCTGATCGCCGGGGCTGGCGCC 150
HF4  GCGGCCTCGCCGCCCTCACGGCCCTCGGCCTGATCGCCGGGGCTGGCGCC 150

GIL1 CTCACCGCCCCCGCCGCGCAGGCCGCCGACGCCGCAGCTCCCGCCGGCAT 200
HF4  CTCACCGCCCCCGCCGCGCAGGCCGCCGACGCCGCAGCTCCCGCCGGCAT 200

GIL1 CGGATCCGACTTCGAATCCGGCACGGGCGCATGGGCGCCGCGCGGGGACG 250
HF4  CGGATCCGACTTCGAATCCGGCACGGGCGCATGGGCGCCGCGCGGGGACG 250

GIL1 GCGTCCGCATCGCCCCGAGTACCGAGGCGCGCACCGGATCCGGCAGCCTG 300
HF4  GCGTCCGCATCGCCCCGAGTACCGAGGCGCGCACCGGATCCGGCAGCCTG 300

GIL1 CTCGTCACCGACCGGACGCAGGAGTGGCACGGCGCCGCGCTGGACGTCAC 350
HF4  CTCGTCACCGACCGGACGCAGGAGTGGCACGGCGCCGCGCTGGACGTCAC 350

GIL1 GGGCGCGCTGACCGTCGGCCAGCAGGTCGAGGTCACGGTGTGGGCGCGCC 400
HF4  GGGCGCGCTGACCGTCGGCCAGCAGGTCGAGGTCACGGTGTGGGCGCGCC 400

GIL1 TGGCCGCGGGGGAGGAGCCCGCATCCCTCAAGGTCTCCGTGCAGCGCGAC 450
HF4  TGTCCGCGGGGGAGGAGCCCGCATCCCTCAAGGTCTCCGTGCAGCGCGAC 450
       *

GIL1 ACCGGCGGCGGCAGCGGCTACGACGGCGTGGCGGGAGCCGCCGCGCGCGT 500
HF4  ACCGGCGGCGGCAGCGGCTACGACGGCGTGGCGGGAGCCGCCGCGCGCGT 500

GIL1 CACCGCCGACGCCTGGACGGAGCTCACGGGCACGTACACGCTCGGCGGCC 550
HF4  CACCGCCGACGCCTGGACGGAGCTCACGGGCACATACACGCTCGGCGGCC 550
                                      *

GIL1 CGGTCGACAAGGCGCAGGTGTACGTGGAGGGGACCGTCGGCGCGGACTTC 600
HF4  CGGTCGACAAGGCGCAGGTGTACGTGGAGGGGACCGTCGGCGCGGACTTC 600

GIL1 CTCCTCGACGACTTCCAGTTGGGCGAGGCCGTTTCCACGCCCGTGCAGAC 650
HF4  CTCCTCTACGACTTCCAGTTGGGCGAGGCCGTTTCCACGCCCGTGCAGAC 650
           *

GIL1 CGACATCCCGGCGCTCCAGGACGTCCTCGGCGCCCGCGGCATCGAGCACG 700
HF4  CGACATCCCGGCGCTCCAGGACGTCCTCGGCGCCCGCGGCATCGAGCACG 700

GIL1 TGGGCGTCGCGATCGACGGCCGCGAGACGGTCGGCGCCGGGGCGGATCTG 750
HF4  TGGGCGTCGCGATCGACGGCCGCGAGACGGTCGGCGCCGGGGCGGATCTG 750
```

```
GIL1 GTGGGTCGGCAGTTCAACGCGTTCACGCCCGAGAACGCCGGGAAGCCCGA 800
HF4  GTGGGTCGGCAGTTCAACGCGTTCACGCCCGAGAACGCCGGGAAGCCCGA 800

GIL1 GAGCGTCCAGCCGGAGGAGGGCCGCTTCACGTTCGCGCAGATCGACCAGC 850
HF4  GAGCGTCCAGCCGGAGGAGGGCCGCTTCACGTTCGCGCAGATCGACCAGC 850

GIL1 TGCTCGACTACGCCGACAGGACCGGCACGAAGGTCTACTACCACGTGCTG 900
HF4  TGCTCGACTACGCCGACAGGACCGGCACGAAGGTCTACTACCACGTGCTG 900

GIL1 TTCTGGCACTCGCAGACCCCCGCGTGGTTCTTCCTCGACGGCGACCGGCC 950
HF4  TTCTGGCACTCGCAGACCCCCGCGTGGTTCTTCCTCGACGGCGACCGGCC 950

GIL1 GCTCACCGACAGCCCCGCCGACCAGGCGCTGCTCCGGGCGCGCATGGAGG 1000
HF4  GCTCACCGACAGCCCCGCCGACCAGGCGCTGCTCCGGGCGCGCATGGAGG 1000

GIL1 CGCACGTGAAGGGCATCGCGGACCACGTGGCGGCCCGCTACCCCGACGGC 1050
HF4  CGCACGTGAAGGGCATCGCGGACCACGTGGCGGCCCGCTACCCCGACGGC 1050

GIL1 GGCAGCCCGATCTGGGCGATGGACGTCGTGAACGAGGTCATCGACGACGG 1100
HF4  GGCAGCCCGATCTGGGCGATGGACGTCGTGAACGAGGTCATCGACGACGG 1100

GIL1 CCCCAACGACAACGCGCACGACATGCGCGACAGCCGCTGGTACCAGGTGC 1150
HF4  CCCCAACGACAACGCGCACGACATGCGCGACAGCCGCTGGTACCAGGTGC 1150

GIL1 TGGGGGAGGGCTTCGTCGACGAGGGCTTCCGCCTCGCGCGCGCGTACTTC 1200
HF4  TGGGGGAGGGCTTCGTCGACGAGGGCTTCCGCCTCGCGCGCGCGTACTTC 1200

GIL1 CCGGGGGTGAAGCTCTTCATCAACGACTACAACACGGAGCTGCCCACGAA 1250
HF4  CCGGGGGTGAAGCTCTTCATCAACGACTACAACACGGAGCTGCCCACGAA 1250

GIL1 GCGCGCCGACTACCTCGAGCTGATCTCCGCCCTCGTGGCCCGCGGTGTGC 1300
HF4  GCGCGCCGACTACCTCGAGCTGATCTCCGCCCTCGTGGCCCGCGGTGTGC 1300

GIL1 CGATCGACGGGGTGGGCCACCAGGCGCACGTCGACTTCGCCCGGCCCGTC 1350
HF4  CGATCGACGGGGTGGGCCACCAGGCGCACGTCGACTTCGCCCGGCCCGTC 1350

GIL1 TCGTGGCTGCGCGACTCCATCCGCGCGGTCGAGCGCATCGACACGCGCCT 1400
HF4  TCGTGGCTGCGCGACTCCATCCGCGCGGTCGAGCGCATCGACACGCGCCT 1400

GIL1 GCTGCAGGCGATCACCGAGCTCGACGTGAACGCGTCGAACCAGAACGAGG 1450
HF4  GCTGCAGGCGATCACCGAGCTCGACGTGAACGCGTCGAACCAGAACGAGG 1450

GIL1 GCGCGGACGTGAGCGGCGCTCCGCAGGATCCGTACACGCCCGTGTACGCG 1500
HF4  GCGCGGACGTGAGCGGCGCTCCGCAGGATCCGTACACGCCCGTGTACGCA 1500
                                                    *

GIL1 GACGACGCCGACGCGGGCGCCGAGGTCGGGCACTACTACCGCGACCTCTT 1550
```

```
HF4  GACGACGCCGACGCGGGCGCCGAGGTCGGGCACTACTACCGCGACCTCTT 1550

GIL1 CCAGATGATCCGCCAGCAGGCCGCGTCCATCGACTCGGTCACCTTCTGGG 1600
HF4  CCAGATGATCCGCCAGCAGGCCGCGTCCATCGACTCGGTCACCTTCTGGG 1600

GIL1 GCGTGAGCAACGCGCGCAGCTGGCTGCGCACGTGGCCGATCGCGCGTCCG 1650
HF4  GCGTGAGCAACGCGCGCAGCTGGCTGCGCACGTGGCCGATCGCGCGTCCG 1650

GIL1 TGGGAGCAGCCGCTGCCGTTCGACGACGACCTGCAGGCGGCTCCCGCCTA 1700
HF4  TGGGAGCAGCCGCTGCTGTTCGACGACGACCTGCAGGCGGCTCCCGCCTA 1700
                     *

GIL1 CTGGGGCATCGTCGACGCGAAGCGCCTCCCGGCACGACCGGCCGACCTCT 1750
HF4  CTGGGGCATCGTCGACGCGAAGCGCCTCCCGGCACGACCGGCCGACCTCT 1750

GIL1 CGGCGCCGCGCATCGCGGACGTCGACGACATCACGGCGGTCGCGACGAAG 1800
HF4  CGGCGCCGCGCATCGCGGACGTCGACGACATCACGGCGGTCGCGACGAAG 1800

GIL1 GCAGCGGGGGTCCGCGTGCCGTACGCGCTGCCCTCGGCGATCGACACGCG 1850
HF4  GCAGCGGGGGTCCGCGTGCCGTACGCGCTGCCCTCGGCGATCGACACGCG 1850

GIL1 CGACGGCAACCTGCGCGTGCTGTGCGCGCCGCCCCGCAGCGGGATCTTCC 1900
HF4  CGACGGCAACGTGCGCGTGCTGTGCGCGCCGCCCCGCAGCGGGATCTTCC 1900
               *

GIL1 CCGTCGGCACCACCACGGTCACCTGCACCGCGAAGGACCGCGCCGGCAAC 1950
HF4  CCGTCGGCACCACCACGGTCACCTGCACCGCGAAGGACCGCGCCGGCAAC 1950

GIL1 GTCCGGACGAGCGAGTTCGACGTGATCGTGACGCGCGCCGGCTCGTGAC 1999
HF4  GTCCGGACGAGCGAGTTCGACGTGATCGTGACGCGCGCCGGCTCGTGAC 1999
```

```
Cmn02654_Pectate_lyase
GIL1 ATGCGCATCCGCCTCCTCCCCGCCGCGCTCGCCGGCGCGCTCCTGCTCGT 50
HF4  ATGCGCATCCGCCTCCTCCCCGCCGCGCTCGCCGGCGCGCTCCTGCTCGT 50

GIL1 CGCGCTGACGGGCACCGGCCCGACCGCTCCCGCCAGCGCCGCGACCGCGA 100
HF4  CGCGCTGACGGGCACCGGCCCGACCGCTCCCGCCAGCGCCGCGACCGCGA 100

GIL1 GCGGCACCTCCGCCACCGCGAGCGCGTCCGCCGACTCCGGCTCCGGCTCC 150
HF4  GCGGCACCTCCGCCACCGCGAGCGCGTCCGCCGACTCCGGCTCCGGCTCC 150

GIL1 GCGGCCACGGGCGCCGGCCCCACCGCCCGCGTCGCCAGCGTCGCCGCCCT 200
HF4  GCGGCCACGGGCGCCGGCCCCACCGCCCGCGTCGCCAGCGTCGCCGCCCT 200

GIL1 GCGCGACGCCATCGCGCAGCACGTGCACCACATCGTCATCACCACCTCGC 250
HF4  GCGCGACGCCATCGCGCAGCACGTGCACCACATCGTCATCACCACCTCGC 250

GIL1 TCTACGGCGGCACCTCGCCCGTCTACGTCGACGTGCTCGACTCGGCATCG 300
HF4  TCTACGGCGGCACCTCGCCCGTCTACGTCGACGTGCTCGACTCGGCATCG 300

GIL1 AGCGGCACCGTCATCGAGGGCGCCACCCCGACGGTGGAACTCGTGAACAT 350
HF4  AGCGGCACCGTCATCGAGGGCGCCACCCCGACGGTGGAACTCGTGAACAT 350

GIL1 CCAGCTGCGGATCTTCGACCTCACCTACGCGGGCGCCATCCACGACGTGA 400
HF4  CCAGCTGCGGATCTTCGACCTCACCTACGCGGGCGCCATCCACGACGTGA 400

GIL1 CGGTGCGCGACCTCACCATGCACGGCCGCATCCAGGACCTCGTCGCGCTC 450
HF4  CGGTGCGCGACCTCACCATGCACGGCCGCATCCAGGACCTCGTCGCGCTC 450

GIL1 ACGGGAGCGGCGACGAAGCCCTACGGCGTCGGCGTCAACTACGAGGCCAT 500
HF4  ACGGGAGCGGCGACGAAGCCCTACGGTGTCGGCGTCAACTACGAGGCCAT 500
                               *

GIL1 CAGCGTGCGCGGGGCCGCGCACGTGACGATCGACCACATCGTCGCCTACG 550
HF4  CAGCGTGCGCGGGGCCGCGCACGTGACGATCGACCACATCGTCGCCTACG 550

GIL1 ACACCACCGACGACCTTATGTCGGTGACGCGCGGGGCCGACGACGTCACC 600
HF4  ACACCACCGACGACCTTATGTCGGTGACGCGCGGGGCCGACGACGTCACC 600

GIL1 ATCTCCAACAGCACGTTCTCGTACTCGCGCGCGTACGCGGACATCGACCC 650
HF4  ATCTCCAACAGCACGTTCTCGTACTCGCGCGCGTACGCGGACATCGACCC 650

GIL1 GAGCATCACGTGGGACTGGGGCTTCGGCCTGCAGCCGCTCGCGTCCGAGC 700
HF4  GAGCATCACGTGGGACTGGGGCTTCGGCCTGCAGCCGCTCGCGTCCGAGC 700

GIL1 GCCTCGGGATCCTCGTGGGCGCCAACGCGAAGGACAGCTACGCCGTCACC 750
HF4  GCCTCGGGATCCTCGTGGGCGCCAACGCGAAGGACAGCTACGCCGTCACC 750

GIL1 GGCCACCTCCACGTGAACCTCGTCGACGACCACATCGGCCCGCTCGTCCG 800
```

```
HF4  GGCCACCTCCACGTGAACCTCGTCGACGACCACATCGGCCCGCTCGTCCG 800

GIL1 GGGACGGCCGCTGCTGCGCGGCGACGTGGACGTGAAGGGCGTGACGTTCG 850
HF4  GGGACGGCCGCTGCTGCGCGGCGACGTGGACGTGAAGGGCGTGACGTTCG 850

GIL1 ACAACACGCAGGCAGGATCCGAGCAGTACGCGGCCATCGAGGTCGGCAGC 900
HF4  ACAACACGCAGGCAGGATCCGAGCAGTACGCGGCCATCGAGGTCGGCAGC 900

GIL1 GGCGGCCGGGTCGTCGTCGAGGGCTGCGAGTTCGACCACTCGAACAACCC 950
HF4  GGCGGCCGGGTCGTCGTCGAGGGCTGCGAGTTCGACCACTCGAACAACCC 950

GIL1 CATCGCGATCCACCTCGACTCACCCTCGGACACCTA 986
HF4  CATCGCGATCCACCTCGACTCACCCTCGGACACCTA 986
```
