# Supplementary material for: Characterization and Comparison of Clavibacter michiganensis subsp. nebraskensis Strains Recovered from Epiphytic and Symptomatic Infections of Maize in Iowa
Source: PLoS One. 2015 Nov 24;10(11):e0143553. doi: 10.1371/journal.pone.0143553 (PMC4658045; doi:10.1371/journal.pone.0143553)
Supplement: S2 File — (PDF) [file pone.0143553.s002.pdf]

```
Cmn00144_CEL_A_GENE
NE3 MAVGATFGAFAAPASAATAAGPGSGGSESQPGPAKPGAISAKWQPGGSWA 50
HF4 MAVGATFGAFAAPASAATAAGPGSGGSESQPGPAKPGAISAKWQPGGSWA 50

NE3 SGYVANLDVTATDAVTGWTVSWASPETTGVVNSWGMRCSVASNTVTCTGT 100
HF4 SGYVANLDVTATDAVTGWTVSWASPETTGVVNSWGMRCSVASNTVTCTGT 100

NE3 DWASELAAGQTVSVGVQLAGGPAPSSPQISATAAGTPPSQPTPPSQPTPP 150
HF4 DWASELAAGQTVSVGVQLAGGPAPSSPQISATAAGTPPSQPTPPSQPTPP 150

NE3 SQPTPPSQ------SATHGRATHYSLGTGNTIANGNCSMPAVPADRMYVA 200
HF4 SQPTPPSQPTPPSQSATHGRATHYSLGTGNTIANGNCSMPAVPADRMYVA 200
            ******

NE3 VSSPEYSGAAACGSYLLVTGPKGTVRVQIVDQCHECEIGHLDLSEEAFRA 250
HF4 VSSPEYSGAAACGSYLLVTGPKGTVRVQIVDQCHECEIGHLDLSEEAFRA 250

NE3 VGDFDAGVIPISYTTVRDPDVPDVAVRVKEGSSRWWAGLQILNAGNPIDH 300
HF4 VGDFDAGVIPISYTTVRDPDVPDVAVRVKEGSSRWWAGLQILNAGNPIDH 300

NE3 VEVQADGQWLGLSRTDYGYWVTPSPIPDGPMTVRVTDQYGRSVVLPGIRM 350
HF4 VEVQADGQWLGLSRTDYGYWVTPSPIPDGPMTVRVTDQYGRSVVLPGIRM 350

NE3 APGEIQSTARRFYPVHZ 367
HF4 APGEIQSTARRFYPVHZ 367
```

```
Cmn02650_Endoglucanase_gene
GIL1 MQHHRHPIRTATTLVLGLALTAGILTAASPASAATTGPAPTVASGAPTVS 50
HF4  MQHHRHPIRTATTLVLGLALTAGILTAASPASAATTGPAPTVASAAPTVS 50
                                                  *

GIL1 DARLRFGVATPGGPTDTGELDAVAQQVGEDPSIVLAYADFTQAPPIAALD 100
HF4  DARLRFGVATPGGPTDTGELDAVAQQVGEDPSIVLAYADFTQAPPIAALD 100

GIL1 SVAARGAETLLTWEPWTAGAGVDQPTFTNASIAAGDHDAYIREWGAALAK 150
HF4  SVAARGAETLLTWEPWTAGAGVDQPTFTNASIAAGDHDAYIREWGAALAK 150

GIL1 WGGPVSLRYAHEMNGDWYPWADGVNGNAPGSYAAAFRHVHDVVVGAGATN 200
HF4  WGGPVSLRYAHEMNGDWYPWADGVNGNAPGSYAAAFRHVHDVVVGAGATN 200

GIL1 VRWVWTPNVPYTGSTALAGLYPGAGYVDVVGLDGYNWGSVAGQSWTAPSD 250
HF4  VRWVWTPNVPYTGSTALAGLYPGAGYVDVVGLDGYNWGSVAGQSWTAPSD 250

GIL1 LFGAGLEQLRAIAPGKPIVIAETASSEVGGSKAQWDGELVSFLQAQTDVV 300
HF4  LFGAGLEQLRAIAPGKPIVIAETASSEVGGSKAQWDGELVSFLQAQTDVV 300

GIL1 AFVWFDMDKEADWRIGSSASSATAIHDALAARRLQZ 336
HF4  AFVWFDMDKEADWRIGSSASSATAIHDALAARRLQZ 336
```

```
Cmn02651_Endoglucanase_gene
NE3 MPTRRRLKCAVVTAVAPAVVTATVLVAPAAGARAASAQAAPTAPAATVLS 50
HF4 MPTRRRLKCAVVTAVAPAVVTATVLVAPAAGARAASAQAAPTAPAATVLS 50

NE3 VASGPVGTPVTVTGTGFPAKKAAVVAVGSTTKRITTTATGRFTTAITIPR 100
HF4 VASGPVGTPVTVTGTGFPAKKAAVVAVGSTTKRITTTATGRFTTAITIPR 100

NE3 TSLSTIRITATAGTRSAGAPFTVTTAKSNGSARLGASAPAPTTTPAPAPT 150
HF4 TSLSTIRITATAGTRSAGAPFTVTTAKSNGSARLGASAPA---------- 140
                                            **********

NE3 GAPTSTAAPAPATSSPAPAASAAPAISSARLRLGLSTPGGPTANGELDAA 200
HF4 ------------TSSPAPAASAAPAISSARLRLGLSTPGGPTANGELDAA 178
    ************

NE3 STTLGESPSIVMSHVDFTHPAPIAGLRSVAARGADSLITWEPWQGGAGVD 250
HF4 STTLGESPSIVMSHVDFTHPAPIAGLRSVAARGADSLITWEPWQGGAGVD 228

NE3 QPAYANARIVAGDYDSYIRSWGADLAKYGQPVYLRFGHEMNGNWYPWSDG 300
HF4 QPAYANARIVAGDYDSYIRSWGADLAKYGQPVYLRFGHEMNGNWYPWSDG 278

NE3 VNGNASGSYVQAWKHVHDLVAAQGATNVKWVWSPNVPYPGSTDLASLYPG 350
HF4 VNGNASGSYVQAWKHVHDLVAAQGATNVKWVWSPNVPYPGSTDLASLYPG 328

NE3 ADQVDVVALDGYNWGAVPGQRWTAPADLFGPGMAQLRAVAPGKPLIIGEV 400
HF4 ADQVDVVALDGYNWGAVPGQRWTAPADLFGPGMAQLRAVAPGKPLIIGEV 378

NE3 ASGETGGSKADWDRDLVAYLQSQPDVLGFVWFDFKKEEDWRIDSSAASAT 450
HF4 ASGETGGSKADWDRDLVAYLQSQPDVLGFVWFDFKKEEDWRIDSSAASAT 428

NE3 ALRDALALRRGZ 462
HF4 ALRDALALRRGZ 440
```

XylanaseB_GENE

```
GIL1 MSIPRTDTSALADVSPPRLGRSRFAPRPRRDRRRGLAALTALGLIAGAGA 50
HF4  MSIPRTDTSALADVSPPRLGRSRFAPRPRRDRRRGLAALTALGLIAGAGA 50

GIL1 LTAPAAQAADAAAPAGIGSDFESGTGAWAPRGDGVRIAPSTEARTGSGSL 100
HF4  LTAPAAQAADAAAPAGIGSDFESGTGAWAPRGDGVRIAPSTEARTGSGSL 100

GIL1 LVTDRTQEWHGAALDVTGALTVGQQVEVTVWARLAAGEEPASLKVSVQRD 150
HF4  LVTDRTQEWHGAALDVTGALTVGQQVEVTVWARLSAGEEPASLKVSVQRD 150
                                       *

GIL1 TGGGSGYDGVAGAAARVTADAWTELTGTYTLGGPVDKAQVYVEGTVGADF 200
HF4  TGGGSGYDGVAGAAARVTADAWTELTGTYTLGGPVDKAQVYVEGTVGADF 200

GIL1 LLDDFQLGEAVSTPVQTDIPALQDVLGARGIEHVGVAIDGRETVGAGADL 250
HF4  LLYDFQLGEAVSTPVQTDIPALQDVLGARGIEHVGVAIDGRETVGAGADL 250
       *

GIL1 VGRQFNAFTPENAGKPESVQPEEGRFTFAQIDQLLDYADRTGTKVYYHVL 300
HF4  VGRQFNAFTPENAGKPESVQPEEGRFTFAQIDQLLDYADRTGTKVYYHVL 300

GIL1 FWHSQTPAWFFLDGDRPLTDSPADQALLRARMEAHVKGIADHVAARYPDG 350
HF4  FWHSQTPAWFFLDGDRPLTDSPADQALLRARMEAHVKGIADHVAARYPDG 350

GIL1 GSPIWAMDVVNEVIDDGPNDNAHDMRDSRWYQVLGEGFVDEGFRLARAYF 400
HF4  GSPIWAMDVVNEVIDDGPNDNAHDMRDSRWYQVLGEGFVDEGFRLARAYF 400

GIL1 PGVKLFINDYNTELPTKRADYLELISALVARGVPIDGVGHQAHVDFARPV 450
HF4  PGVKLFINDYNTELPTKRADYLELISALVARGVPIDGVGHQAHVDFARPV 450

GIL1 SWLRDSIRAVERIDTRLLQAITELDVNASNQNEGADVSGAPQDPYTPVYA 500
HF4  SWLRDSIRAVERIDTRLLQAITELDVNASNQNEGADVSGAPQDPYTPVYA 500

GIL1 DDADAGAEVGHYYRDLFQMIRQQAASIDSVTFWGVSNARSWLRTWPIARP 550
HF4  DDADAGAEVGHYYRDLFQMIRQQAASIDSVTFWGVSNARSWLRTWPIARP 550

GIL1 WEQPLPFDDDLQAAPAYWGIVDAKRLPARPADLSAPRIADVDDITAVATK 600
HF4  WEQPLLFDDDLQAAPAYWGIVDAKRLPARPADLSAPRIADVDDITAVATK 600
          *

GIL1 AAGVRVPYALPSAIDTRDGNLRVLCAPPRSGIFPVGTTTVTCTAKDRAGN 650
HF4  AAGVRVPYALPSAIDTRDGNVRVLCAPPRSGIFPVGTTTVTCTAKDRAGN 650
                         *

GIL1 VRTSEFDVIVTRAGSZ 666
HF4  VRTSEFDVIVTRAGSZ 666
```

```
Cmn02654_Pectate_lyase
GIL1 MRIRLLPAALAGALLLVALTGTGPTAPASAATASGTSATASASADSGSGS 50
HF4  MRIRLLPAALAGALLLVALTGTGPTAPASAATASGTSATASASADSGSGS 50

GIL1 AATGAGPTARVASVAALRDAIAQHVHHIVITTSLYGGTSPVYVDVLDSAS 100
HF4  AATGAGPTARVASVAALRDAIAQHVHHIVITTSLYGGTSPVYVDVLDSAS 100

GIL1 SGTVIEGATPTVELVNIQLRIFDLTYAGAIHDVTVRDLTMHGRIQDLVAL 150
HF4  SGTVIEGATPTVELVNIQLRIFDLTYAGAIHDVTVRDLTMHGRIQDLVAL 150

GIL1 TGAATKPYGVGVNYEAISVRGAAHVTIDHIVAYDTTDDLMSVTRGADDVT 200
HF4  TGAATKPYGVGVNYEAISVRGAAHVTIDHIVAYDTTDDLMSVTRGADDVT 200

GIL1 ISNSTFSYSRAYADIDPSITWDWGFGLQPLASERLGILVGANAKDSYAVT 250
HF4  ISNSTFSYSRAYADIDPSITWDWGFGLQPLASERLGILVGANAKDSYAVT 250

GIL1 GHLHVNLVDDHIGPLVRGRPLLRGDVDVKGVTFDNTQAGSEQYAAIEVGS 300
HF4  GHLHVNLVDDHIGPLVRGRPLLRGDVDVKGVTFDNTQAGSEQYAAIEVGS 300

GIL1 GGRVVVEGCEFDHSNNPIAIHLDSPSDT 328
HF4  GGRVVVEGCEFDHSNNPIAIHLDSPSDT 328
```
