# Supplementary material for: Characterization and Comparison of Clavibacter michiganensis subsp. nebraskensis Strains Recovered from Epiphytic and Symptomatic Infections of Maize in Iowa
Source: PLoS One. 2015 Nov 24;10(11):e0143553. doi: 10.1371/journal.pone.0143553 (PMC4658045; doi:10.1371/journal.pone.0143553)
Supplement: S1 Table — (DOCX) [file pone.0143553.s003.docx]

**S1 Table. GPS coordinates of nearest town to which fields are located.**

| **Field Number** | **County, State** | **Nearest town** | **GPS co-ordinates** | |
| --- | --- | --- | --- | --- |
|  |  |  | **Latitude** | **Longitude** |
| 1 | Iowa, IA | Williamsburg | 41.664281 | -92.012334 |
| 2 | Boone, IA | Boone | 42.061127 | −93.886057 |
| 3 | Story, IA | Gilbert | 42.105858 | -93.645185 |
| 4 | Story, IA | Gilbert | 42.105858 | -93.645185 |
| 5 | Grant, NE | Grant | 40.844405 | -101.726109 |
| 6 | Carroll, IA | Glidden | 42.058263 | -94.728359 |
| 7 | Story, IA | Gilbert | 42.105858 | -93.645185 |
| 8 | Boone, IA | Boone | 42.061127 | −93.886057 |
| 9 | Boone, IA | Boone | 42.061127 | −93.886057 |
